# Visualizing structural dynamics during nuclease activation across evolutionarily diverse IscB and CRISPR-Cas9 enzymes

**DOI:** 10.21203/rs.3.rs-10202707/v1

**Published:** 2026-09-08

**Authors:** Osamu Nureki, Kosuke Onishi, Yutaro Shuto, Josh Müller, Mikihiro Shibata, Satoshi Omura

**Affiliations:** University of Tokyo; University of Tokyo; The University of Tokyo; Faculty of Medicine, University of Bern; Kanazawa University; University of Tokyo

## Abstract

Cas9 nucleases of CRISPR-Cas adaptive immune systems are programmable RNA-guided DNA endonucleases that evolved from the transposon-associated IscB enzymes. Comparative structural studies have revealed substantial architectural elaboration during this evolutionary transition, including the replacement of the larger ωRNA scaffold of IscB with the REC domains of Cas9, capable of recognizing longer guide–target heteroduplexes. However, these evolutionary insights remain restricted to static structural features, and the dynamic properties that underlie nuclease activation have not been systematically explored across the IscB-to-Cas9 lineage. Here, by combining cryo-electron microscopy structural analysis with real-space, real-time high-speed atomic force microscopy imaging, we visualize the stepwise nuclease activation mechanisms of evolutionarily diverse IscB and Cas9 nucleases, including IscB.m13, type II-B PsCas9, type II-C CjCas9, and type II-D Cas9d MG34-1. We show here that the IscB family employs two distinct strategies for HNH autoinhibition, with one representing the direct evolutionary precursor of the Cas9 nuclease activation mechanism. Moreover, although the stacking-based protein–DNA interaction that stabilizes an intermediate state along the activation pathway is conserved in both IscB and Cas9 enzymes, Cas9 has acquired an additional gating element that prevents premature HNH activation immediately before catalysis. Together with previously reported structures of OgeuIscB and SpCas9, these analyses establish an atlas of the structural dynamics underlying nuclease activation across the IscB-to-Cas9 lineage, uncovering both conserved and diversified activation mechanisms and substantially advancing the mechanistic and evolutionary understanding of these RNA-guided endonucleases.

## Introduction

Cas9 is a programmable RNA-guided DNA endonuclease that executes the interference step of the type II CRISPR-Cas adaptive immune system, which defends prokaryotes against invading mobile genetic elements^1-3^. In complex with a dual RNA guide consisting of CRISPR RNA (crRNA) and trans-activating crRNA (tracrRNA), or with an engineered single guide RNA (sgRNA), Cas9 binds and cleaves double-stranded DNA (dsDNA) targets complementary to the crRNA spacer sequence^3,4^. In addition to guide–target complementarity, Cas9 requires a protospacer-adjacent motif (PAM) immediately downstream of the target site for DNA recognition. Phylogenetic analyses have divided type II CRISPR-Cas systems into four subtypes (II-A to II-D), distinguished primarily by the size and domain architecture of the Cas9 protein^5,6^. Among the diverse Cas9 orthologs, the type II-A enzyme from *Streptococcus pyogenes* (SpCas9) is notable for its potent cleavage activity and has become the dominant platform for genome editing and related molecular applications^7,8^.

IscB, an RNA-guided DNA endonuclease encoded by IS200/IS605 transposons and constituting the catalytic component of the OMEGA (obligate mobile element-guided activity) system, has been proposed as the evolutionary precursor of Cas9 on the basis of comparative sequence analyses^9,10^. Like Cas9, IscB associates with a single RNA guide, designated the ωRNA, and cleaves dsDNA targets complementary to the ωRNA guide segment in the presence of a specific transposon/target-adjacent motif (TAM) immediately downstream of the target sequence^10^. Structural analyses have revealed that, despite substantial differences in the protein and guide RNA sizes, Cas9 (~1,600 aa) and IscB (~500 aa) share core architectural features^11,12^. Both enzymes adopt a bilobed architecture composed of recognition (REC) and nuclease (NUC) lobes connected by a bridge helix (BH), accommodate the guide–target heteroduplex within the groove between these lobes, and possess HNH and RuvC nuclease domains that cleave the target strand (TS) and non-target strand (NTS), respectively. A growing body of structural studies further indicates that the molecular evolution from IscB to Cas9 has involved several stepwise events, such as the loss of the IscB-specific N-terminal PLMP domain, the acquisition of a zinc-finger-containing REC3 domain, the extension of the BH and the REC1 domain, and the further expansion of REC domains, which have replaced the ωRNA scaffold to allow longer target recognition^13^. However, these evolutionary insights remain limited to static structural features and do not yet encompass the dynamic properties that underlie the nuclease activity of these enzymes.

The traditional view of proteins as adopting a single native structure has long been replaced by the modern understanding that the native state comprises an ensemble of conformations^14^. Within this ensemble framework, proteins sample diverse conformations through local fluctuations, such as loop motions and side-chain rotations, as well as through global rearrangements involving relative movements of domains. Such dynamic behavior is exemplified by the IscB and Cas9 nucleases, whose activation is accompanied by extensive domain rearrangements. Single-molecule Förster resonance energy transfer (FRET) analyses have revealed allosteric coupling among the REC2, REC3, and HNH domains during the activation of SpCas9^15-17^, and high-speed atomic force microscopy (HS-AFM) imaging has directly visualized real-space, real-time fluctuations of the Cas9 HNH domain^18,19^. More recently, cryo-electron microscopy (cryo-EM) studies have resolved the stepwise nuclease activation pathways of OgeuIscB and SpCas9 from the early to late stages of R-loop formation^20,21^, providing detailed mechanistic insights into their modes of action. Nevertheless, comparable analyses of R-loop formation across evolutionarily diverse IscB and Cas9 nucleases are lacking, leaving the evolution of the dynamic properties of these enzymes largely uncharacterized.

Here, by combining cryo-EM structural analysis with real-space, real-time HS-AFM imaging, we comprehensively visualize the stepwise nuclease activation mechanisms of evolutionarily diverse IscB and Cas9 nucleases, including IscB.m13^22^, type II-B PsCas9^23^, type II-C CjCas9^24^, and type II-D Cas9d MG34-1^25^, providing an atlas of the structural dynamics underlying nuclease activation across this lineage. This atlas reveals both the conserved and diversified domain rearrangements during nuclease activation, extending the evolutionary view of the IscB-to-Cas9 transition from static architectures to protein dynamics.

## Results

### IscB.m13 regulates nuclease activation through a minimal ωRNA scaffold and guide-anchored HNH autoinhibition

A previous study revealed that, unlike Cas9 nucleases, OgeuIscB utilizes the ωRNA as a lid that occludes both the RuvC (guide RNA-lid) and HNH (ωRNA-lid) nuclease domains during an early stage of R-loop formation to prevent promiscuous DNA cleavage^20^. Upon R-loop propagation, OgeuIscB adopts an active conformation in which the elongated guide–target heteroduplex mechanically displaces the HNH domain, allowing it to dock onto the TS cleavage site. However, IscB orthologs from different species possess ωRNA scaffolds with markedly divergent secondary structures. Thus, whether the ωRNA-lid-mediated nuclease activation mechanism proposed for OgeuIscB is conserved among other IscB orthologs remains unclear.

To elucidate the stepwise nuclease activation mechanism of an IscB ortholog distinct from OgeuIscB, we focused on IscB.m13, a recently identified ortholog that is phylogenetically divergent from OgeuIscB^22^ (Extended Data Fig. 1a). IscB.m13 comprises 481 amino acid residues, associates with a 211-nt ωRNA scaffold, and recognizes the ATAAA sequence as its TAM. To capture a series of structural snapshots along the nuclease activation pathway of IscB.m13, we designed two target DNA constructs harboring 10- and 18-nt sequences complementary to the ωRNA guide and reconstituted the corresponding IscB.m13–ωRNA–target DNA complexes (Extended Data Fig. 1b). The cryo-EM structures of these complexes revealed three distinct functional states, designated the pre-, early, and late R-loop states, respectively (Fig. 1b-d, Extended Data Fig. 1b–g, and Supplementary Table 1). The domain organization of IscB.m13 resembles that of other IscBs, comprising PLMP, RuvC, HNH, WED, and TI domains connected by the BH, REC linker, and Hinge motifs (Hinges I and II) (Fig. 1a). By contrast, the IscB.m13 ωRNA scaffold consists of three stem loops (the guide-adaptor hairpin, terminal hairpin 1, and terminal hairpin 2) and four stems (the nexus stem, central stem, nexus pseudoknot (PK) hairpin, and nexus PK) (Fig. 1d), and lacks the stem loops 2 and 3 that, in OgeuIscB, anchor the HNH domain in an inactive conformation by serving as an ωRNA lid (Fig. 1e-g). These structural observations suggest that IscB.m13 regulates nuclease activation through a distinct mechanism enabled by its minimal ωRNA scaffold, as detailed below.

In the pre-R-loop state, the TAM-containing DNA duplex is bound to IscB.m13 and gripped by the WED and TI domains, whereas the ωRNA guide remains unhybridized with the target DNA (Fig. 1b-d). Instead, we observed a continuous density corresponding to the ωRNA guide nucleotides G(−11) to C(−1), of which G(−9) to C(−1) are pre-ordered in an A-form geometry and poised for heteroduplex formation, whereas G(−11) and U(−10) are flipped out, suggesting that nine nucleotides serve as a seed in IscB.m13 (Fig. 1b-d). Notably, the HNH domain, together with Hinges I and II, adopts an inactive conformation in which the HNH active site is located away from the potential TS cleavage site and the RuvC active site is occluded by Hinge I. Although this autoinhibited arrangement parallels that of OgeuIscB, it is achieved through a distinct mechanism. In OgeuIscB, the inactive configuration of the HNH domain is established through interactions between the HNH domain and the nexus PK of the ωRNA, together with hydrophobic contacts between Hinge I and the RuvC domain, such that the HNH active site is occluded by the ωRNA-lid (Fig. 1h-j). In IscB.m13, by contrast, neither interaction is observed, owing to the shorter ωRNA scaffold and the altered local structure of Hinge I (Fig. 1h-j). Instead, the inactive conformation of IscB.m13 is achieved by interactions between the HNH domain and the ωRNA guide, in which Tyr288 of Hinge II stacks onto the flipped-out guide nucleotide G(−11) (Fig. 1k). These structural observations suggest that, unlike OgeuIscB, IscB.m13 lacks the ωRNA scaffold element that serves as a lid to prevent premature HNH activation and instead employs the ωRNA guide to anchor the HNH domain and Hinge motifs in an inactive conformation.

In the early R-loop state, the guide–target heteroduplex has formed, yet the HNH domain remains inactive (Fig. 1b-d). Notably, although the target DNA construct carried a 10-nt sequence complementary to the ωRNA guide, the heteroduplex extended to only 9 base pairs. Leu291 of the inactive Hinge II forms a hydrophobic interaction with the heteroduplex and sterically precludes its extension beyond 9 base pairs, while Tyr288 still stacks onto the flipped-out guide nucleotide G(−11) to stabilize the flipped guide conformation (Fig. 1k). This early R-loop state likely represents a kinetic intermediate in which the heteroduplex is provisionally stabilized before further propagation. Through this Hinge II-mediated stabilization, IscB.m13 discriminates against severely mismatched substrates while remaining tolerant of partial mismatches, allowing the engagement of a broader range of target sequences^21^.

In the late R-loop state, we observed a density corresponding to a 15-bp guide–target heteroduplex, whereas the upstream ωRNA guide and target DNA are disordered, likely owing to their conformational flexibility (Fig. 1b). The TAM-distal region of the heteroduplex is exposed to the solvent without protein contacts and takes the position previously occupied by the inactive HNH domain, thereby disordering the HNH domain. This observation suggests that, although IscB.m13 does not directly contact the TAM-distal heteroduplex, the elongated guide–target heteroduplex can stably propagate and mechanically displace the HNH domain to promote catalysis, as observed for OgeuIscB. We did not obtain a structure of the active conformational state in which the HNH domain is stably docked onto the TS cleavage site. This raises the possibility that, in IscB.m13, the HNH domain does not adopt a stably docked state at the TS cleavage site but instead transiently and repeatedly samples the active position, enabling stochastic TS cleavage. Collectively, these structural observations indicate that, unlike OgeuIscB, IscB.m13 lacks the ωRNA-lid and instead employs the ωRNA guide for HNH autoinhibition, followed by stochastic target cleavage by the flexible HNH domain (Supplementary Movie S1).

### The minimal REC1 and REC3 domains of Cas9d.34-1 drive stepwise nuclease activation through sequential conformational checkpoints

Recent studies identified the type II-D Cas9 nucleases, which are characterized by compact molecular sizes and may represent potential evolutionary intermediates between IscB and larger Cas9 enzymes^10,13,25,26^. Among them, Cas9d MG34-1 (hereafter Cas9d.34-1) comprises 747 amino acids, which is much smaller than other Cas9s, and exhibits modest but detectable genome-editing activity in human cells, thereby highlighting its potential as a compact genome-editing platform^25^. Although a previous study reported the cryo-EM structure of the Cas9d.34-1–sgRNA–target DNA complex, the RuvC and HNH domains are poorly resolved^27^, leaving the structural basis for the nuclease activation of compact Cas9d.34-1 unclear. To capture a series of structural snapshots during the nuclease activation of Cas9d.34-1, we designed partially matched DNA targets containing 10-, 15-, and 20-nt complementary sequences toward the sgRNA guide and reconstituted the Cas9d.34-1–sgRNA–target DNA complexes (Extended Data Fig. 2a). We then determined the cryo-EM structures of the Cas9d.34-1–sgRNA–target DNA complex in seven distinct functional states (Fig. 2a-d, Extended Data Fig. 2, and Supplementary Table 2). These states were broadly classified according to the length of the guide–target heteroduplex and designated as the early, middle, and late R-loop states. The overall architecture of Cas9d.34-1 is largely consistent with that of the previously reported Cas9d.34-1 structure, consisting of the REC lobe (REC1 and REC3 domains) and the NUC lobe (RuvC, HNH, WED, and PI domains, as well as L1 and L2 linkers) connected by the BH (Fig. 2a). Notably, the ωRNA scaffold found in IscB is replaced in Cas9d.34-1 by the minimal REC1 and REC3 domains, which regulate stepwise R-loop formation through domain rearrangements, while a central stem-like structure derived from the IscB ωRNA is retained, tethering both the guide–target heteroduplex and the relative positions of the REC1 and REC3 domains (Figs. 1c and 2c).

In the early R-loop state, Cas9d.34-1 accommodates the 10-bp guide–target heteroduplex within the positively charged central channel between the REC and NUC lobes (Fig. 2c and Extended Data Fig. 3a). The REC3 domain, together with the HNH and RuvC domains that were poorly resolved in the previously reported Cas9d.34-1 structure, lines the central channel, with the HNH domain positioned in an inactive conformation away from the TS cleavage site. Whereas IscB tethers the HNH domain in an inactive position through the ωRNA, Cas9d.34-1 instead achieves this inactivation through interactions between the L2 linker and the HNH domain (Extended Data Fig. 3b). In addition, this inactive domain arrangement leaves sufficient space within the channel to accommodate further extension of the guide–target heteroduplex beyond 10 base pairs (Extended Data Fig. 3a), indicating that the early R-loop state represents an intermediate state poised for subsequent R-loop propagation.

From the dataset obtained using the partially matched target DNA substrate containing a 15-nt guide-complementary sequence, we identified three middle R-loop substates, designated middle R-loop states I–III (Fig. 2b, 2c and Extended Data Fig. 3a). These substates differ in both the length of the guide–target heteroduplex and the conformational flexibility of the NUC lobe. In the middle R-loop state-I, the REC3 domain shifts inward toward the guide–target heteroduplex and adopts a closed conformation. Notably, His244 in the REC3 domain stacks onto dA13 of the TS, thereby preventing further heteroduplex formation beyond 13 base pairs (Extended Data Fig. 3c). The His244 stacking interaction thus stabilizes this intermediate state, enabling Cas9d.34-1 to discriminate against severely mismatched substrates while remaining tolerant of partial mismatches that may arise in escape variants of phage DNA. In the middle R-loop state-II, in contrast, the REC3 and HNH domains cooperatively undergo outward movement to provide an expanded groove that accommodates the extended guide–target heteroduplex beyond 13 base pairs (Extended Data Fig. 3d). Within this groove, we observed continuous density into which a 19-bp guide–target heteroduplex could be modeled in an A-form geometry, despite the use of a target containing only a 15-nt guide-complementary sequence (Extended Data Fig. 3e). This indicates that nucleotides G(−19) to A(−16) of the sgRNA guide form pseudo-base pairs with dA15 to dG19 of the TS in the absence of complementarity. The 19-bp heteroduplex was also observed in the middle R-loop state-III, in which the RuvC and HNH domains are entirely disordered as observed in the previous study, suggesting that the longer heteroduplex formation releases the nuclease domains of Cas9d.34-1 to facilitate subsequent R-loop formation (Fig. 2b, 2c and Extended Data Fig. 3a). Collectively, these structural observations suggest that Cas9d.34-1 regulates R-loop formation at the 13-bp guide–target heteroduplex to monitor PAM-proximal guide–target complementarity. Once heteroduplex formation proceeds beyond 13 base pairs, R-loop propagation can spontaneously advance to approximately 19 base pairs, accompanied by disengagement of the RuvC and HNH domains to allow further R-loop formation.

In the late R-loop states, where a 20-bp guide–target heteroduplex is formed, we identified three different conformational substates based on the HNH domain arrangement, and designated them as late R-loop states I–III (Fig. 2b, 2c, and Extended Data Fig. 3a). In the late R-loop state-I, the PAM-distal dsDNA target is located at the position occupied by the HNH domain in the early and middle R-loop states. Accordingly, the HNH domain moves inward and docks onto the RuvC domain, thereby avoiding steric clashes with the PAM-distal target dsDNA while preventing premature cleavage of the NTS before the completion of R-loop formation (Extended Data Fig. 3f). Thus, the HNH domain acts as a structural gatekeeper that regulates the transition from the middle to late R-loop state. Subsequently, in the late R-loop state-II, the displaced NTS approaches the RuvC active site and pushes the HNH domain away from the RuvC domain, thus disordering the HNH domain along with the L1 and L2 linkers (Extended Data Fig. 3g). In the late R-loop state-III, the displaced NTS is bound to the RuvC active site, where it is coordinated by two magnesium ions. In addition, the HNH domain docks onto the TS cleavage site with a single magnesium ion, indicating that the late R-loop state-III represents the catalytically active state of Cas9d.34-1. In this active state, the L1 linker engages the major groove of the PAM-distal guide–target heteroduplex and is positioned near the L2 linker through hydrophobic interactions, thereby stabilizing the active conformation (Extended Data Fig. 3h). Notably, the docking of the HNH domain onto the TS is accompanied by the outward movement of a helix in the REC1 domain (residues 112–134; hereafter referred to as the REC1 lid), suggesting that the REC1 lid provides a final checkpoint by regulating HNH-domain docking before full nuclease activation (Extended Data Fig. 3i). Taken together, these observations indicate that Cas9d.34-1 undergoes stepwise R-loop propagation through sequential conformational checkpoints, in which the minimal REC1 and REC3 domains that substitute for the IscB ωRNA play central roles (Supplementary Movie S2). The 13-bp guide–target heteroduplex serves as an intermediate checkpoint for guide–target complementarity, whereas the subsequent rearrangements of the REC3, RuvC, and HNH domains and the REC1 lid couple further R-loop propagation to complete the catalysis.

### The flexible REC3 domain triggers an open-to-closed conformational transition that activates CjCas9

CjCas9 is a type II-C Cas9 nuclease comprising 984 amino acid residues. Its compact size, which is compatible with the limited cargo capacity of AAV vectors, together with its robust genome-editing activity *in vivo*, makes CjCas9 a particularly attractive platform for *in vivo* gene therapy^24^. Although we previously reported the crystal structure of the CjCas9–sgRNA–target DNA complex, the crystallization construct lacked the HNH domain as well as the L1 and L2 linkers^28^. Therefore, the stepwise mechanism underlying nuclease activation in full-length CjCas9 has remained elusive. To address this gap, we determined the cryo-EM structures of the full-length CjCas9–sgRNA–target DNA complex in four distinct functional states at overall resolutions of 2.6–3.0 Å using target DNA substrates containing 10-, 15-, and 22-nt guide-complementary sequences, which we designated as the early, middle, and late R-loop states, respectively (Fig. 3a-d, Extended Data Fig. 4 and Supplementary Table 3). CjCas9 has larger REC1 and REC3 domains compared to Cas9d.34-1 but lacks the central stem-like structure observed in the IscB ωRNA. In addition, the HNH domain and L1 and L2 linkers, which were absent from the previous crystal structure, are resolved in the middle and late R-loop states, thereby revealing the complete architecture of CjCas9 (Fig. 3b).

In the early R-loop state, CjCas9 accommodates the 10-bp guide–target heteroduplex by gripping the PAM-proximal region with the BH and expanded REC1 domain (Extended Data Fig. 5a and b). Notably, the entire REC3 and HNH domains along with the L1 and L2 linkers are completely disordered in the density map, indicating their intrinsic flexibility in early R-loop formation (Fig. 3b). Accordingly, the PAM-distal portion of the guide–target heteroduplex is exposed to the solvent and lacks stable protein contacts, suggesting that CjCas9 does not strictly constrain the trajectory of the heteroduplex at the initial stage of R-loop formation. To directly visualize the conformational dynamics inferred from the disordered density in this state, we performed HS-AFM observations, which enable real-space, real-time visualization of single molecules. The CjCas9–sgRNA–target DNA complex was pre-assembled using a target DNA substrate containing a 10-nt guide-complementary sequence and applied to an AFM substrate for HS-AFM observations. Consistent with the cryo-EM structure of CjCas9 in the early R-loop state, two globular domains exhibiting dynamic motion on the DNA-bound complex were observed (Fig. 3e, 3f and Supplementary Movie S3). Based on AFM simulations of the cryo-EM structure of CjCas9 and inferences from the DNA orientation, these two flexible domains are identified as the REC3 and HNH domains, respectively (Fig. 3e). In addition, the HS-AFM observations revealed that the bound dsDNA maintained a linear conformation, indicating that CjCas9 does not bend the target DNA at this stage, consistent with the absence of stable protein contacts along the PAM-distal heteroduplex (Fig. 3e and Supplementary Movie S3). These observations suggest that the early R-loop state of CjCas9 is intrinsically dynamic, characterized by the flexible REC3 and HNH domains and an unconstrained target DNA.

In the middle R-loop state, the extension of the guide–target heteroduplex to 15 base pairs establishes contacts with the REC3 domain, thereby promoting the ordering of the REC3 domain and the accommodation of the elongated heteroduplex within the central channel (Fig. 3b). The HNH domain and the L1 and L2 linkers also become ordered, with the HNH domain adopting an inactive position far from the scissile phosphate of the TS. Notably, Gln490 in the L1 linker forms a hydrogen bond with the ribose moiety of G(−11), indicating that R-loop propagation beyond 11 base pairs cooperatively stabilizes the inactive HNH conformation through interactions between the sgRNA guide and L1 linker (Extended Data Fig. 5c). Moreover, the HNH domain docks onto the RuvC domain to occlude the RuvC active site, likely preventing premature cleavage of the NTS before full R-loop formation (Extended Data Fig. 5c). These findings suggest that the middle R-loop state represents an intermediate checkpoint in which heteroduplex extension promotes REC3 ordering, while maintaining both the HNH and RuvC nuclease domains in inactive configurations.

As observed for Cas9d.34-1, CjCas9 also adopts two distinct conformational states upon the formation of the full 22-bp guide–target heteroduplex, which differ primarily in the arrangement of the HNH domain and are designated late R-loop state-I and -II, respectively (Fig. 3b-d). In the late R-loop state-I, the REC3 domain shifts inward toward the heteroduplex, which enables stable interactions between the REC3 domain and the PAM-distal region of the guide–target heteroduplex (Fig. 3g). The PAM-distal target DNA forms a B-form duplex downstream of the 22-bp guide–target heteroduplex, and this PAM-distal DNA duplex would sterically clash with the L2 linker in the middle R-loop conformation, dissociating and disordering the HNH domain along with the L1 and L2 linkers (Fig. 3h). The cleavage site of the TS is occluded by a segment within the REC1 domain (residues 138–154; referred to as the REC1 lid) to prevent the dissociated HNH domain from docking onto the cleavage site of the TS, indicating that the late R-loop state-I represents the final checkpoint before full nuclease activation (Fig. 3i). In the late R-loop state-II, the REC1 lid becomes disordered, exposing the TS cleavage site, and the HNH domain becomes ordered and docks onto the TS cleavage site with a single coordinated magnesium ion, indicating that the late R-loop state II represents the catalytically active state (Fig. 3j). Notably, the REC3 domain moves further inward, accompanied by a substantial downward shift of the guide–target heteroduplex (Extended Data Fig. 5d). This downward shift positions the PAM-distal region of the heteroduplex for engagement by the L1 linker, thereby enabling the L1 linker to adopt an active conformation stabilized by multiple electrostatic interactions with the heteroduplex (Extended Data Fig. 5e). HS-AFM observations of the late R-loop CjCas9 complex, using a target DNA bearing a 22-nt guide-complementary sequence (Supplementary Movie S4), revealed two notable features consistent with the cryo-EM structures. The REC3 domain aligned rigidly with the target DNA, and we detected a pronounced bend in the target DNA, in contrast to the linear conformation observed in the early R-loop HS-AFM analysis (Extended Data Fig. 5f), corresponding to the conformational distortion captured in the cryo-EM structures of the late R-loop state. Together, these findings indicate that, in CjCas9, the REC3 domain remains disordered during the early phase of R-loop formation, allowing relatively unconstrained heteroduplex propagation, whereas its subsequent open-to-closed conformational transition drives the downward shift of the guide–target heteroduplex (Supplementary Movie S5). This REC3-driven heteroduplex repositioning serves as a structural trigger for the coordinated rearrangements of the L1 linker, HNH domain, and REC1 lid, ultimately enabling full nuclease activation.

### The REC3 clamp provides two intermediate checkpoints for precise catalysis by PsCas9

PsCas9 is a type II-B Cas9 nuclease that mediates robust and accurate DNA cleavage both *in vitro* and *in vivo*^23^. Although the cryo-EM structure of PsCas9 in the product state has provided important insights into its DNA recognition mechanism^29^, the stepwise process by which PsCas9 achieves precise catalysis through nuclease activation remains unclear. To clarify the structural basis of stepwise nuclease activation in PsCas9, we determined the PsCas9–sgRNA–target DNA complex structures in four distinct functional states at overall resolutions ranging from 2.7 to 3.3 Å (Fig. 4a-d and Extended Data Fig. 6). The four structures are primarily distinguished by the guide–target heteroduplex lengths of 7, 13, and 22 base pairs, which we designated as the early, middle, and late R-loop states, respectively (Fig. 4b-d). PsCas9 contains expanded REC domains relative to Cas9d.34-1, including an additional REC2 domain (residues 101–210) inserted within the REC1 domain, and a distinctive REC3 clamp (residues 514–610) insertion within the REC3 domain that is highly conserved across type II-B Cas9 nucleases^30,31^ (Fig. 4b).

In the early R-loop state, PsCas9 adopts a closed conformation in which the REC3 clamp extensively interacts with the L1 linker and the HNH domain, stabilizing the HNH domain in an inactive conformation located far from the scissile phosphate of the TS (Fig. 4e). In addition, a helix and loop motif (residues 599–617, referred to as the REC3 gating motif) within the REC3 clamp protrudes into the central channel between the REC and NUC lobes, thereby sterically blocking the extension of the guide–target heteroduplex beyond 7 base pairs (Fig. 4f). Moreover, we observed an ambiguous density corresponding to the PAM-distal dsDNA within a positively charged cleft formed by the REC2 and REC3 domains (Fig. 4g). Consistent with this closed architecture captured by cryo-EM, HS-AFM observations revealed that the PsCas9 complex harboring a 10-bp guide–target heteroduplex maintains a comparatively rigid conformation throughout the early R-loop state over the imaging time course, exhibiting limited domain fluctuations relative to CjCas9 (Fig. 4h and Supplementary Movie S6). Collectively, these observations suggest that the early R-loop state, defined by a 7-bp guide–target heteroduplex, constitutes an initial checkpoint for target interrogation, while simultaneously accommodating the PAM-distal target DNA duplex within the PsCas9 complex in preparation for subsequent heteroduplex propagation.

In the middle R-loop state, the REC3 clamp undergoes an outward displacement, allowing the formation of the 13-bp guide–target heteroduplex (Fig. 4i). Notably, the helix of the REC3 gating motif adopts a loop conformation to accommodate the elongated heteroduplex, while Tyr606 within this motif stacks onto A(−13) in the sgRNA (Fig. 4j), thereby preventing further heteroduplex propagation beyond 13 base pairs. These findings suggest that the closed-to-open rearrangement of the REC3 clamp and the helix-to-loop transition of the REC3 gating motif cooperatively regulate heteroduplex elongation from 7 to 13 base pairs, with the Tyr606 stacking interaction stabilizing a structurally distinct intermediate at the 13-bp length. Consistent with this interpretation, a previous study showed that PsCas9 nuclease activation is particularly sensitive to mismatches at positions 6-13 relative to the PAM^23^. Together with the earlier 7-bp intermediate, these two partitioned intermediates provide PsCas9 with additional opportunities to discriminate against mismatched substrates, accounting for its higher fidelity relative to other Cas9 enzymes.

In the late R-loop state, in which the 22-bp guide–target heteroduplex is fully formed, we identified two distinct conformational substates that differ primarily in the arrangement of the HNH domain, which we designated late R-loop state-I and -II, respectively (Fig. 4b and 4c). In the late R-loop state-I, the REC3 gating motif shifts outward to again adopt a helical conformation, creating sufficient space for the completion of the fully matched 22-bp heteroduplex (Fig. 4k). The PAM-distal target DNA forms an ideal B-form duplex downstream of the guide–target heteroduplex, and this PAM-distal duplex would sterically clash with the HNH domain in its inactive position, thereby likely promoting the dissociation and increased conformational flexibility of the HNH domain, consistent with its disorder in the cryo-EM density map (Extended Data Fig. 7a). In addition, the REC2 domain sterically occludes the TS cleavage site to prevent the HNH domain from docking onto the TS, suggesting that the late R-loop state-I serves as a third checkpoint just before the activation. The overall structure of the late R-loop state-II is almost identical to that of the late R-loop state-I, except that the HNH domain undergoes a rotational movement and docks onto the cleavage site of the TS with single-coordinated magnesium ions, indicating that the late R-loop state-II represents a catalytically active state (Fig. 4b and 4c). This HNH activation is accompanied by an outward movement of the REC2 domain, which creates the necessary space for the HNH domain to access the DNA cleavage site (Extended Data Fig. 7b). Taken together, these findings suggest that, in PsCas9, the REC3 clamp, which is highly conserved in type II-B Cas9s, governs the early phase of stepwise R-loop propagation through a closed-to open conformational transition, whereas the REC2 domain acts at the final checkpoint of R-loop formation by regulating the transition of the HNH domain from an inactive to active conformation (Supplementary Movie S7).

### The dynamic basis of nuclease activation in evolutionarily diverse IscB and Cas9 nucleases

Our series of structural snapshots, together with previously reported structures of OgeuIscB^20^ and SpCas9^21^ captured along their nuclease activation pathways, provide an atlas of the structural dynamics underlying nuclease activation across the evolutionarily diverse IscB and Cas9 enzymes (types II-A to II-D) (Fig. 5). Within the ancestral IscB family, the structural comparison delineates at least two modes of HNH autoinhibition that prevent promiscuous DNA cleavage during early R-loop formation, differing in the presence or absence of an ωRNA-lid (Fig. 5). OgeuIscB represents the ωRNA-lid mode, in which the nexus PK hairpin and stem loop 2 anchor the HNH domain in an inactive position and occlude its active site, respectively (Fig. 1i). These ωRNA-lid elements undergo dynamic structural rearrangements during nuclease activation in OgeuIscB, analogous to the REC domains of Cas9 enzymes. By contrast, IscB.m13 possesses a minimal ωRNA scaffold that lacks these stem loops and instead employs the ωRNA guide to maintain the HNH domain in an inactive conformation, with little or no conformational rearrangement of the ωRNA observed during nuclease activation (Fig. 1i). Notably, TbaIscB, an evolutionary intermediate along the IscB-to-Cas9 trajectory, appears to retain an ωRNA lid analogous to that of OgeuIscB^13^, suggesting that ωRNA-lid-mediated regulation likely represents the ancestral state from which Cas9 nucleases evolved REC domains for activation control. In both OgeuIscB and IscB.m13, the inactive HNH domain is subsequently activated by extension of the guide–target heteroduplex. We propose that, once activated, the HNH domain does not adopt a stably docked conformation at the TS cleavage site but instead transiently and repeatedly attaches to the active position to cleave the TS stochastically, consistent with the disordered HNH domain in all reported IscB structures other than activity-enhanced variants^11-13,20,26^.

A common structural feature underlying R-loop propagation across the IscB-to-Cas9 evolution is a steric checkpoint, imposed by a hydrophobic or aromatic residue that stacks onto the guide–target heteroduplex to halt its extension at a defined length (Fig. 5). In OgeuIscB, Phe152 stacks onto the sixth base pair of the guide–target heteroduplex, while in IscB.m13, Leu291 of the Hinge II sterically precludes heteroduplex extension beyond 9 base pairs, defining the early R-loop checkpoint in each enzyme. Similarly, in Cas9d.34-1, His244 of the REC3 domain stacks onto dA13 of the TS to enforce a checkpoint at 13 base pairs, while in PsCas9, Tyr606 of the REC3 gating motif stacks onto A(−13) of the sgRNA. A previous study also revealed that, in SpCas9, Tyr450 of the REC1 domain stacks onto the sixth guide–target base pair^21^, indicating that hydrophobic and aromatic stacking onto the heteroduplex represents an evolutionarily conserved strategy for interrogating PAM-proximal guide–target complementarity and stabilizing the intermediate checkpoint to enhance reaction fidelity, which is retained from the ancestral IscB lineage through the elaborated Cas9 enzymes. This shared architecture discriminates against severely mismatched substrates while tolerating partial mismatches arising in escape variants of phage DNA. Although the corresponding checkpoint state is not captured in our series of CjCas9 structures, Phe239 of the CjCas9 REC1 domain occupies a position analogous to that of Tyr450 in SpCas9, suggesting that CjCas9 also employs a stacking-based regulatory mechanism in the earlier phase of R-loop formation.

In contrast to these conserved features, the transition from IscB to Cas9 is marked by a fundamental shift in the molecular basis of HNH autoinhibition, achieved through two distinct layers of regulation acquired in the Cas9 lineage (Fig. 5). First, whereas IscBs anchor the HNH domain through the ωRNA, either as a dedicated ωRNA-lid (OgeuIscB) or via the ωRNA guide (IscB.m13), Cas9 nucleases universally employ the L1 and L2 linkers to tether the HNH domain in an inactive conformation (Fig. 5). Second, Cas9 nucleases have acquired a dedicated protein element that occludes the TS cleavage site to prevent premature HNH docking, a regulatory layer absent from IscBs. In Cas9d.34-1 and CjCas9, this role is performed by the REC1 lid, a segment of the REC1 domain that physically blocks the cleavage site until late in R-loop propagation, while in PsCas9 and SpCas9, the role is fulfilled by the acquired REC2 domain (Fig. 5). The convergence of two architecturally distinct elements onto the same gatekeeping function underscores its importance for preventing off-target cleavage in the larger Cas9 enzymes. This additional gating likely became necessary as Cas9 nucleases evolved away from the stochastic TS-sampling mode of IscBs and toward a stably docked active state, in which premature HNH engagement with the TS would risk off-target cleavage. Together, these two regulatory layers represent defining features of activation control that accompanied the evolution of Cas9 from its IscB ancestor.

A comparison of the nuclease activation mechanisms of Cas9 enzymes from four different subtypes further reveals that they fall into two mechanistic classes, based on the directionality of REC-domain rearrangement during activation (Fig. 5). Type II-D Cas9d.34-1 and type II-B PsCas9 follow a closed-to-open trajectory, in which the REC domains that were initially closed onto the heteroduplex open to permit R-loop propagation. Type II-A SpCas9 and type II-C CjCas9 instead follow the reverse, open-to-closed trajectory, in which an initially disordered or open REC3 domain closes onto the extended heteroduplex to trigger activation. Notably, previous studies have reported that PsCas9 and Cas9d are more sensitive to mismatches than SpCas9^23,32^. This observation suggests that the closed conformation adopted during the early or middle stages of R-loop formation in PsCas9 and Cas9d.34-1 imposes a defined steric barrier that requires comparatively more accurate base pairing to overcome, accounting for the higher mismatch sensitivity of these enzymes. By contrast, the open-to-closed class permits relatively unconstrained early R-loop propagation and establishes a defined checkpoint, involving close protein–DNA contacts, only at late stages, thereby accommodating a broader range of guide–target pairings before activation. Taken together, our structural comparison reveals that, despite the conserved checkpoint-gated activation mechanism, the IscB-to-Cas9 evolution has diversified along multiple axes, including the molecular basis of HNH autoinhibition, the acquisition of dedicated gating elements at the TS cleavage site, and the directionality of REC-domain rearrangement, providing a structural framework for understanding how mismatch sensitivity and activation kinetics have been progressively tuned across the family.

## Discussion

Recent structural studies have revealed the overall architectures of IscB and various Cas9 nucleases in single states along the nuclease activation pathway, shedding light on their molecular evolution and showing that the larger ωRNA scaffold of IscB has been progressively replaced by the REC domains of Cas9 to support the recognition of longer guide–target heteroduplexes^11-13^. However, beyond these static architectures, the dynamic properties responsible for the nuclease function, such as domain rearrangements during activation, are themselves expected to be conserved through molecular evolution^14^, yet only fragmentary studies have systematically addressed the stepwise activation mechanisms of IscB and Cas9 enzymes^20,21,33^. The dynamic basis that has been conserved or diversified along the evolutionary trajectory from IscB to Cas9 has therefore remained largely unresolved. In this study, by integrating cryo-EM analysis along the nuclease activation pathway with single-molecule HS-AFM imaging, we comprehensively visualize the stepwise activation process across evolutionarily diverse IscB and Cas9 nucleases, providing evolutionary insights into the protein dynamics that have been retained or acquired across this lineage.

A key feature conserved across the nuclease activation pathways of IscB and Cas9 enzymes is the hydrophobic interaction between a hydrophobic or aromatic residue and the PAM (TAM)-proximal guide–target heteroduplex in an early phase of R-loop formation. This shared interaction stabilizes an intermediate state along the activation pathway, allowing these enzymes to discriminate against severely mismatched targets while tolerating partial mismatches that may arise during target sequence diversification. This adaptive property has likely supported the retention of this mechanism throughout the evolution of RNA-guided nucleases from the transposon-associated IscBs to the Cas9 enzymes of adaptive immunity. Notably, a recent study reported that the alanine substitution of Tyr450 in SpCas9, the stacking residue that stabilizes the intermediate checkpoint state, enhances the target specificity by exacerbating the destabilization of off-target intermediates, thereby promoting the dissociation of off-target substrates before catalysis^21^. Disrupting the analogous interactions that stabilize the intermediate checkpoint states identified in this study may therefore yield high-fidelity variants of each enzyme, offering a structural strategy for engineering safer Cas9-based genome editing tools with reduced off-target sites, though further functional validation will be required.

Our series of cryo-EM structures also highlights the nuclease activation mechanisms acquired during the evolution of Cas9 from IscB. A notable example is the dedicated gating element that regulates HNH-domain docking onto the TS cleavage site. In IscBs, the HNH domain does not stably engage the TS cleavage site and remains structurally disordered, transiently sampling the active position to execute stochastic DNA cleavage. By contrast, Cas9 enzymes have acquired the capacity to stably dock the HNH domain at the TS cleavage site for more efficient catalysis, consistent with their role as adaptive immune effectors that must rapidly recognize and efficiently cleave invading foreign DNA. Coupled with this transition to stable HNH docking, Cas9 enzymes have universally acquired a dedicated gating element that controls HNH access to the TS cleavage site immediately before full nuclease activation, presumably to prevent the off-target cleavage caused by premature HNH activation. This role is fulfilled by the REC1 lid in type II-C CjCas9, type II-D Cas9d.34-1, and the compact type II-A SaCas9^34^, and by the acquired REC2 domain in type II-A SpCas9 and type II-B PsCas9. The convergence of two architecturally distinct elements on the same gatekeeping function underscores the importance of fidelity control in Cas9-mediated DNA cleavage.

In conclusion, our integrated structural and dynamic analyses establish a unified evolutionary framework for appreciating how the activation mechanisms of RNA-guided nucleases have been both conserved and diversified across the IscB-to-Cas9 lineage. These insights provide a foundation for understanding the basic biology of RNA-guided endonucleases and engineering them for genome editing applications.

## Methods

### Ethical statements

This study did not involve human participants, animal subjects, or any material requiring ethical approval.

### Phylogenetic analysis of IscB orthologs

The amino acid sequences of 408 IscB orthologs were retrieved from Kannan et al. (2025)^26^, to which the IscB.m13 sequence reported by Xiao et al. (2025)^22^ was appended, yielding a final dataset of 409 sequences. Multiple sequence alignment was performed using MAFFT v7.526 under the iterative refinement strategy FFT-NS-i^35^. The resulting alignment, spanning 2,607 positions, was subsequently trimmed using ClipKIT v2.11.4 in the default smart-gap mode^36^, which excised poorly aligned and highly divergent positions, reducing the alignment to 1,806 parsimony-informative positions (30.7% of sites removed). Maximum-likelihood phylogenetic inference was then conducted using IQ-TREE v3.0.1^37,38^, with the optimal substitution model (Q.PFAM+F+R8) selected by ModelFinder^39^ according to the Bayesian Information Criterion (BIC). Branch support values were estimated via 1,000 ultrafast bootstrap replicates^40^. The resulting phylogenetic tree was visualized and annotated using iTOL v6^41^.

### Sample preparation of IscB.m13

The genes encoding the ωRNA (nucleotides −18–215) and IscB.m13 (residues 1–481) were codon optimized, synthesized (Eurofin Genomics), and cloned into the pETDuet vector (Novagen). The IscB protein and ωRNA were coexpressed in *Escherichia coli* Rosetta2 (DE3) by induction with 0.1 mM IPTG (Nacalai Tesque) at 20°C for 20 h. The *E. coli* cells were lysed by sonication in buffer (20 mM HEPES-KOH, pH 7.4, 20 mM imidazole, 0.15 M NaCl, and 10% glycerol) and the lysate was clarified by centrifugation at 10,000 g for 20 min. The supernatant was applied to Ni-NTA Superflow resin (QIAGEN) and the IscB–ωRNA complex was eluted with buffer (20 mM HEPES-KOH, pH 7.4, 300 mM imidazole, and 0.15 M NaCl). The eluted fractions were combined and applied to a Superdex 200 Increase 10/300 GL gel filtration column (GE Healthcare) equilibrated with buffer (20 mM HEPES-KOH, pH 7.4, 0.1 M NaCl, 1 mM MgCl_2_, and 1 mM DTT). The peak fractions were collected.

The IscB.m13–ωRNA–target DNA complex was prepared for cryo-EM analysis using the following procedure. The 10- and 18-nucleotide complementary dsDNA substrates were prepared by annealing a 44-nt TS and a 44-nt NTS containing an ATAAA TAM. Each DNA strand contained phosphorothioate modifications within the phosphate backbone around the cleavage site to prevent DNA cleavage. The peak fractions of the IscB.m13–ωRNA complex were mixed with the TS and the NTS (molar ratio, 1:1.2:1.2) and incubated at room temperature for 10 min. The complexes were then purified on a Superdex 200 Increase 10/300 GL gel filtration column equilibrated with buffer (20 mM HEPES-KOH, pH 7.4, 0.1 M NaCl, 1 mM MgCl_2_, and 1 mM DTT). The peak fraction containing the IscB.m13–ωRNA–target DNA complex was concentrated to an absorbance at 260 nm of 19 to 34, using an Amicon Ultra-0.5 centrifugal filter unit (MWCO: 50 kDa).

### Sample preparation of Cas9d.34-1

The gene encoding Cas9d.34-1 (residues 1–747) was cloned into the pE-SUMO vector with an N-terminal His_6_-tag. The protein was expressed in *Escherichia coli* Rosetta2 (DE3) by induction with 0.1 mM IPTG at 20°C for 20 h. The *E. coli* cells were collected by centrifugation and lysed in buffer A (20 mM Tris-HCl, pH 8.0, 1 M NaCl, and 20 mM imidazole) by sonication. The clarified lysate was applied to Ni-NTA Superflow resin and the protein was eluted with buffer (20 mM Tris-HCl, pH 8.0, 300 mM imidazole, and 0.3 M NaCl). The eluted fractions were applied to a HiTrap Heparin column (Cytiva), equilibrated with buffer (20 mM Tris-HCl, pH 8.0, 0.3 M NaCl). The bound protein was eluted with a linear gradient of 0.3-2 M NaCl and further purified on a HiLoad 16/600 Superdex 200 pg column (GE Healthcare) equilibrated with buffer (20 mM Tris-HCl, pH 8.0, 0.5 M NaCl, 2 mM MgCl_2_, and 1 mM DTT). The peak fractions were collected and stored at −80°C until use. The gRNA was transcribed *in vitro* using T7 RNA polymerase and PCR-amplified double-stranded DNA templates. The resulting gRNA was purified by 10% denaturing (7 M urea) polyacrylamide gel electrophoresis and stored at −20°C until use.

The Cas9d.34-1–sgRNA–target DNA complex was prepared for cryo-EM analysis using the following procedure. The 10-, 15-, and 20-nucleotide complementary dsDNA substrates were prepared by annealing a 41-nt TS and a 41-nt NTS containing a TGG PAM. Each DNA strand contained phosphorothioate modifications within the phosphate backbone around the cleavage site to prevent DNA cleavage. The purified Cas9d.34-1 was mixed with the sgRNA and the target DNA (molar ratio, 1:1.1:1.2) and incubated at 37°C for 30 min. The complexes were then purified on a Superdex 200 Increase 10/300 GL gel filtration column equilibrated with buffer (20 mM Tris-HCl, pH 8.0, 50 mM NaCl, 10 mM MgCl_2_, 10 μM ZnCl_2_, and 1 mM DTT). The peak fraction containing the Cas9d.34–1–sgRNA–target DNA complex was concentrated to an absorbance at 260 nm of 13 to 22, using an Amicon Ultra-0.5 centrifugal filter unit (MWCO: 100 kDa).

### Sample preparation of CjCas9

The gene encoding CjCas9 (residues 1–984) was cloned into the pE-SUMO vector, which encodes an N-terminal His_6_–SUMO tag. The protein was expressed in *Escherichia coli* Rosetta2 (DE3) by induction with 0.1 mM IPTG at 20°C for 20 h. The *E. coli* cells were lysed by sonication in buffer (20 mM HEPES-KOH, pH 7.4, 20 mM imidazole, 1 M NaCl, and 10% glycerol) and the lysate was clarified by centrifugation at 10,000 g for 20 min. The supernatant was applied to Ni-NTA Superflow resin and the protein was eluted with buffer (20 mM HEPES-KOH, pH 7.4, 300 mM imidazole, and 0.3 M NaCl). The eluted fractions were applied to a HiTrap Heparin column (Cytiva), equilibrated with buffer (20 mM HEPES-KOH, pH 7.4, 0.3 M NaCl). The bound protein was eluted with a linear gradient of 0.3-2 M NaCl. The peak fractions were collected and stored at −80°C until use. The sgRNA was transcribed *in vitro* with T7 RNA polymerase, using a partially double-stranded DNA template. The transcribed sgRNA was purified by 8% denaturing urea PAGE, extracted from gel slices with Tris-Borate-EDTA Buffer (Takara), and then ethanol precipitated. The sgRNA pellet was dissolved in Milli-Q water and stored at −20°C.

The CjCas9–sgRNA–target DNA complex was prepared for cryo-EM analysis using the following procedure. The 10-, 15-, and 22-nucleotide complementary dsDNA substrates were prepared by annealing a 44-nt TS and a 44-nt NTS containing an AACAATAC PAM. Each DNA strand contained phosphorothioate modifications within the phosphate backbone around the cleavage site to prevent DNA cleavage. The purified CjCas9 was mixed with the sgRNA and the target DNA (molar ratio, 1:1.2:1.2) and incubated at room temperature for 10 min. The complexes were then purified on a Superdex 200 Increase 10/300 GL gel filtration column equilibrated with buffer (20 mM HEPES-KOH, pH 7.4, 0.1 M NaCl, 1 mM MgCl_2_, and 1 mM DTT). The peak fraction containing the CjCas9–sgRNA–target DNA complex was concentrated to an absorbance at 260 nm of 15 to 34, using an Amicon Ultra-0.5 centrifugal filter unit (MWCO: 100 kDa).

### Sample preparation of PsCas9

The gene encoding PsCas9 (residues 1–1409) was codon optimized, synthesized (Eurofin Genomics), and cloned into the pE-SUMO vector (LifeSensors), which encodes an N-terminal His_6_–SUMO tag. The protein was expressed and purified using the protocol reported previously^42^. Briefly, the protein was expressed in *Escherichia coli* Rosetta2 (DE3) by induction with 0.1 mM IPTG at 20°C for 20 h. The *E. coli* cells were lysed by sonication in buffer (20 mM HEPES-KOH, pH 7.4, 20 mM imidazole, 1 M NaCl, and 10% glycerol) and the lysate was clarified by centrifugation at 10,000 g for 20 min. The supernatant was applied to Ni-NTA Superflow resin and the protein was eluted with buffer (20 mM HEPES-KOH, pH 7.4, 300 mM imidazole, and 0.3 M NaCl). The eluted fractions were applied to a HiTrap Heparin column (Cytiva), equilibrated with buffer (20 mM HEPES-KOH, pH 7.4, 0.3 M NaCl). The bound protein was eluted with a linear gradient of 0.3-2 M NaCl. The peak fractions were collected and loaded onto a HiLoad 16/600 Superdex 200 column (GE Healthcare), equilibrated with buffer (20 mM HEPES-KOH, pH 7.4, 0.5 M NaCl). The peak fractions were collected and stored at −80°C until use. The sgRNA was transcribed *in vitro* with T7 RNA polymerase, using a partially double-stranded DNA template. The transcribed sgRNA was purified by 8% denaturing urea PAGE, extracted from gel slices with Tris-Borate-EDTA Buffer (Takara), and then ethanol precipitated. The sgRNA pellet was dissolved in Milli-Q water and stored at −20°C.

The PsCas9–sgRNA–target DNA complex was prepared for cryo-EM analysis using the following procedure. The 22-nucleotide complementary dsDNA substrate was prepared by annealing a 44-nt TS and a 44-nt NTS containing a TGG PAM. Each DNA strand contained phosphorothioate modifications within the phosphate backbone around the cleavage site to prevent DNA cleavage. The purified PsCas9 was mixed with the sgRNA and the target DNA (molar ratio, 1:1.2:1.2) and incubated at room temperature for 10 min. The complexes were then purified on a Superdex 200 Increase 10/300 GL gel filtration column equilibrated with buffer (20 mM HEPES-KOH, pH 7.4, 0.1 M NaCl, 1 mM MgCl_2_, and 1 mM DTT). The peak fraction containing the PsCas9–sgRNA–target DNA complex was concentrated to an absorbance at 260 nm of 9, using an Amicon Ultra-0.5 centrifugal filter unit (MWCO: 100 kDa).

### Cryo-EM grid preparation and data collection

All grids were glow-discharged, after adding 3 μl amylamine^42^. The IscB.m13–ωRNA–target DNA complexes (A_260_ of 34 and 19) (3 μl) were applied to Au 300-mesh R1.2/1.3 grids (Quantifoil), in a Vitrobot Mark IV at 4°C, with a waiting time of 10 s and a blotting time of 4 s under 100% humidity conditions. The PsCas9–sgRNA–target DNA (A_260_ of 9) (3 μl) was applied to Au 300-mesh R1.2/1.3 grids (Quantifoil), in a Vitrobot Mark IV at 4°C, with a waiting time of 10 s and a blotting time of 4 s under 100% humidity conditions. The CjCas9–sgRNA–target DNA complexes (A_260_ of 34, 24, and 15) were applied to Au 300-mesh R1.2/1.3 grids (Quantifoil), in a Vitrobot Mark IV at 4°C, with a waiting time of 10 s and a blotting time of 4 s under 100% humidity conditions. The grids were then plunge-frozen in liquid ethane, cooled at the temperature of liquid nitrogen.

Micrographs for all datasets were collected with a Titan Krios G3i microscope (Thermo Fisher Scientific) running at 300 kV and equipped with a Gatan Quantum-LS Energy Filter (GIF) and a Gatan K3 Summit direct electron detector in the electron counting mode (The University of Tokyo, Japan). Datasets were collected with a total dose of approximately 50 electrons per Å^2^ per 48 frames by the standard mode, using the EPU software (Thermo Fisher Scientific). The dose-fractionated movies were subjected to beam-induced motion correction and dose weighting using Patch Motion Correction, and the contrast transfer function (CTF) parameters were estimated using Patch-based CTF estimation in cryoSPARC v4.4.0, v4.6.0, v4.7.1, and v5.0.2 ^43,44^.

### Single-particle cryo-EM data processing

Data were processed using cryoSPARC v4.4.0 (20-nt match complexes of Cas9d, and 10-, 15-, 22-nt match complexes of CjCas9), v4.6.0 (22-nt match complexes of PsCas9), v4.7.1 (18-nt match complexes of IscB.m13), and v5.0.2 (10-nt match complexes of IscB.m13)^44^.

For the dataset of 10-nt match IscB.m13, 3,772,093 particles were automatically picked using Template Picker from the 4,452 motion-corrected and dose-weighted micrographs, followed by several rounds of reference-free 2D classification to curate particle sets. The particles were further curated by heterogeneous refinement, using the map derived from cryoSPARC ab initio reconstruction as the template. The best class containing 518,320 particles was subjected to CTF refinement, Reference-Based Motion Correction (RBMC), and 3D classification without alignment^45^. Non-uniform refinement after subsequent postprocessing yielded maps at overall resolutions of 2.80 Å (pre-R-loop state) and 2.60 Å (early R-loop state), according to the Fourier shell correlation (FSC) criterion of 0.143^46,47^. The local resolution was estimated by BlocRes in cryoSPARC.

The datasets for the 18-nt match complexes of IscB.m13, Cas9d.34-1, PsCas9, and CjCas9 were processed using cryoSPARC in a similar manner as described above. For data processing details, see Extended Data Figs. 1,2,4 and 6.

### Model building and validation

The models of IscB.m13 and Cas9d.34.1 were built using the protein model derived from AlphaFold2 as the reference^48^. The models of PsCas9 and CjCas9 were built based on PDB models 8UMF and 5X2G, respectively^28,29^. Model adjustment and nucleic acid building were completed using COOT^49,50^. The models were refined using Servalcat against unsharpened half maps^51^. The reference structure restraints generated by Prosmart were used for the refinement^52^. The stereochemical restraints for phosphorothioate-modified DNA links were generated using AceDRG^53^. The models were validated using MolProbity^54^. Molecular graphics figures were prepared with UCSF ChimeraX 1.11.1^55^.

### HS-AFM observations

HS-AFM observations were conducted using the laboratory-built instrument described previously^56^. The experimental workflow closely followed our earlier study^18^. Briefly, HS-AFM observations were performed in liquid with a cantilever (MNOIC, AC7; 6–7 μm length, 2 μm width, 100 nm thickness) characterized by a spring constant of 0.2 N/m, an approximate resonant frequency near 400 kHz, and a quality factor of 2. Cantilever oscillations were monitored using an optical beam-deflection system in which a 780 nm, 0.8 mW laser was focused onto the cantilever back through a 60× objective lens (Nikon, CFI S Plan Fluor ELWD 60×). Data were collected in the tapping mode, using the “only trace imaging (OTI)” approach to minimize damage from the AFM tip^57^. An approximately 250 nm amorphous carbon tip was deposited onto the original “bird’s beak” probe by electron-beam deposition in an FE-SEM (Verious 5UC, Thermo Fisher Scientific, USA) and subsequently plasma-etching in argon (Tergeo, PIC Scientific, USA) to yield an apex radius of approximately 1 nm. Image acquisition and tip-scanning were managed using the custom UMEX software. For HS-AFM observations, a mica surface was treated for 3 min with 0.00005% (3-aminopropyl)triethoxysilane (APTES) (Sigma-Aldrich). CjCas9 or PsCas9, RNA and DNA were pre-assembled at a 1:1:1 mole ratio (Cas9:RNA:DNA) in AFM imaging buffer. The imaging buffer contained 20 mM Tris-HCl (pH 8.0), 30 mM KCl, 1 mM MgCl_2_, and 10% glycerol. All HS-AFM observations were conducted at room temperature (24–26°C). Each experimental condition was repeated a minimum of three times; displayed images represent a typical example. The ImageJ software was used for image processing and analysis (NIH, USA)^58^. Background subtraction employed a rolling-ball radius of 200 pixels, and noise was reduced with a Gaussian filter (0.5-pixel radius). Frame-to-frame drift was corrected using the Template Matching and Slice Alignment plugin^59^. For tracking the REC3 and HNH domains of CjCas9–sgRNA–DNA complexes, the center positions were determined with the MTrackJ plugin^60^, using Bright centroid snapping and a 15 × 15 pixel^2^ snap window. To simulate the AFM topographies of Cj or PsCas9–sgRNA–DNA, we used BioAFMviewer^61^ to validate the experimentally measured surface profiles. Simulated scans modeled nonelastic collisions between a rigid, conical tip and the protein’s atomic structure represented by its van der Waals surface. Tip parameters were set to an apex sphere radius (R) of 0.5 nm and a cone half-angle (α) of 5.0°.

### Statistics & Reproducibility

No statistical methods were used to predetermine sample size. Sample size was based on experimental feasibility and sample availability.

## Supplementary Material

This is a list of supplementary files associated with this preprint. Click to download.

- cas9dynamicsextendeddatafignsmb.pdf
- cas9dynamicssupplementarytables.pdf
- supplementarymovieS1.mp4
- supplementarymovieS2.mp4
- supplementarymovieS3.mp4
- supplementarymovieS4.mp4
- supplementarymovieS5.mp4
- supplementarymovieS6.mp4
- supplementarymovieS7.mp4

## Figures and Tables

**Figure 1:**
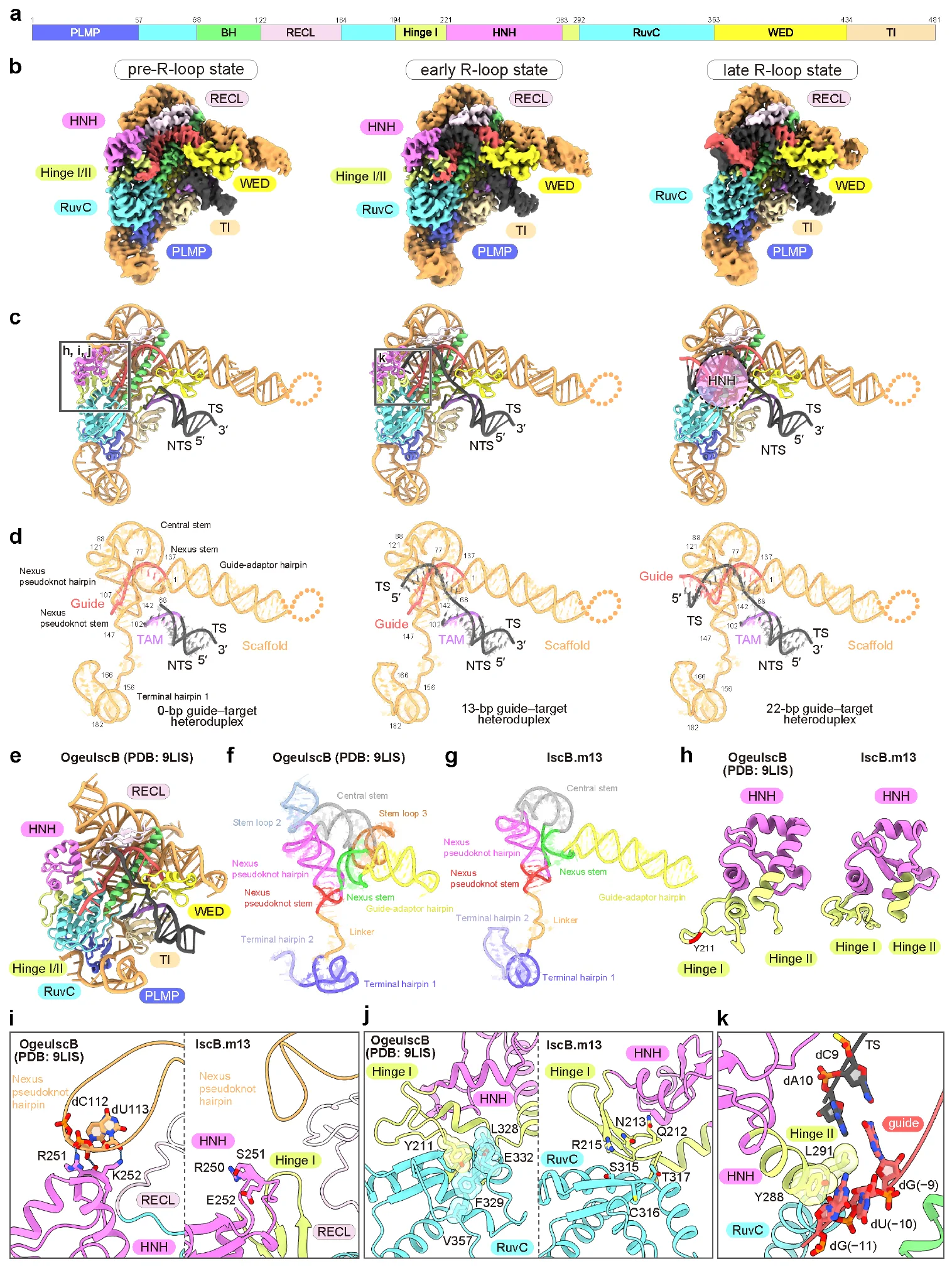
Cryo-EM structures of IscB.m13 in distinct R-loop states. (**a**) Schematic representations of the domain architectures of IscB.m13. (**b and c**) Overview of the pre-, early, and late R-loop states of the IscB.m13–ωRNA–target DNA. Cryo-EM density maps (**b**), and ribbon models (**c**). (**d**) Structures of the ωRNA, the TS, and the NTS. (**e**) Structure of the OgeuIscB–ωRNA–target DNA complex (PDB: 9LIS). (**f and g**) Structures of the ωRNAs of OgeuIscB (**f**) and IscB.m13 (**g**). (**h**) Structural comparison of the Hinge I/II regions and the HNH domains of OgeuIscB and IscB.m13. The red-colored residue makes a hydrophobic contact with the RuvC domain in OgeuIscB. (**i**) Close-up views around the HNH domain and the ωRNA-lid of OgeuIscB (left) and IscB.m13 (right). The HNH domain interacts with the nexus PK of the ωRNA in OgeuIscB, while no interaction is observed in IscB.m13. (**j**) Close-up views around the Hinge I regions and the RuvC domains of OgeuIscB (left) and IscB.m13 (right). Tyr211 in Hinge I forms a hydrophobic interaction with the residues in the RuvC domain. No interaction between Hinge I and RuvC is observed in IscB.m13. (**k**) Close-up view of Hinge II in the early R-loop state of IscB.m13. Tyr288 stacks onto the flipped-out G(−11) of the guide RNA, and Leu291 is inserted between A10 of the DNA target strand and U(−10) of the guide RNA.

**Figure 2:**
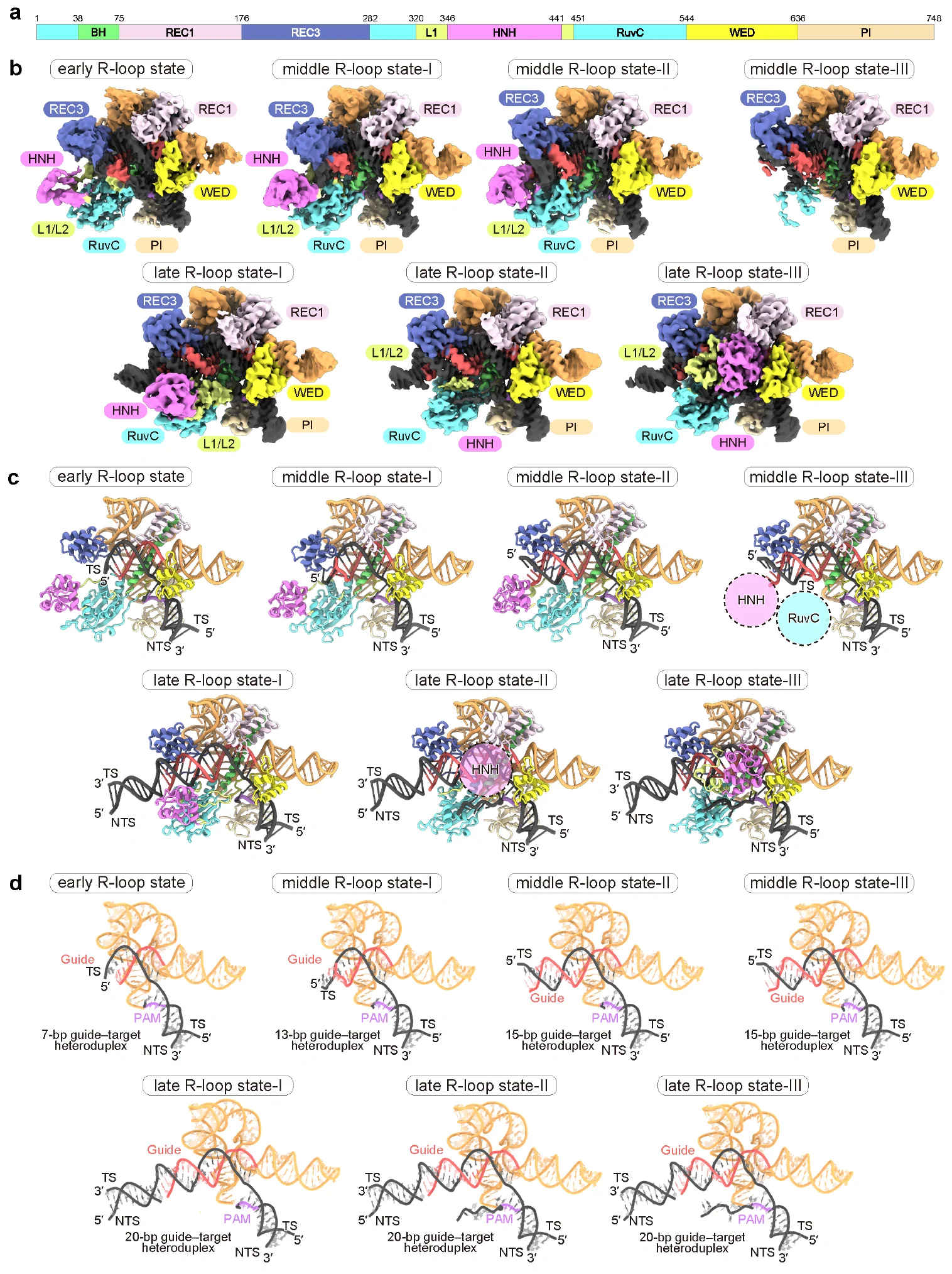
Cryo-EM structures of Cas9d.34-1 in distinct R-loop states. (**a**) Schematic representations of domain architectures of Cas9d.34-1. (**b and c**) Overview of the early, middle-I, middle-II, middle-III, late-I, late-II, and late-III R-loop states of the Cas9d.34-1–sgRNA–target DNA. Cryo-EM density maps (**b**), and ribbon models (**c**). (**d**) Structures of the sgRNA, the TS, and the NTS.

**Figure 3:**
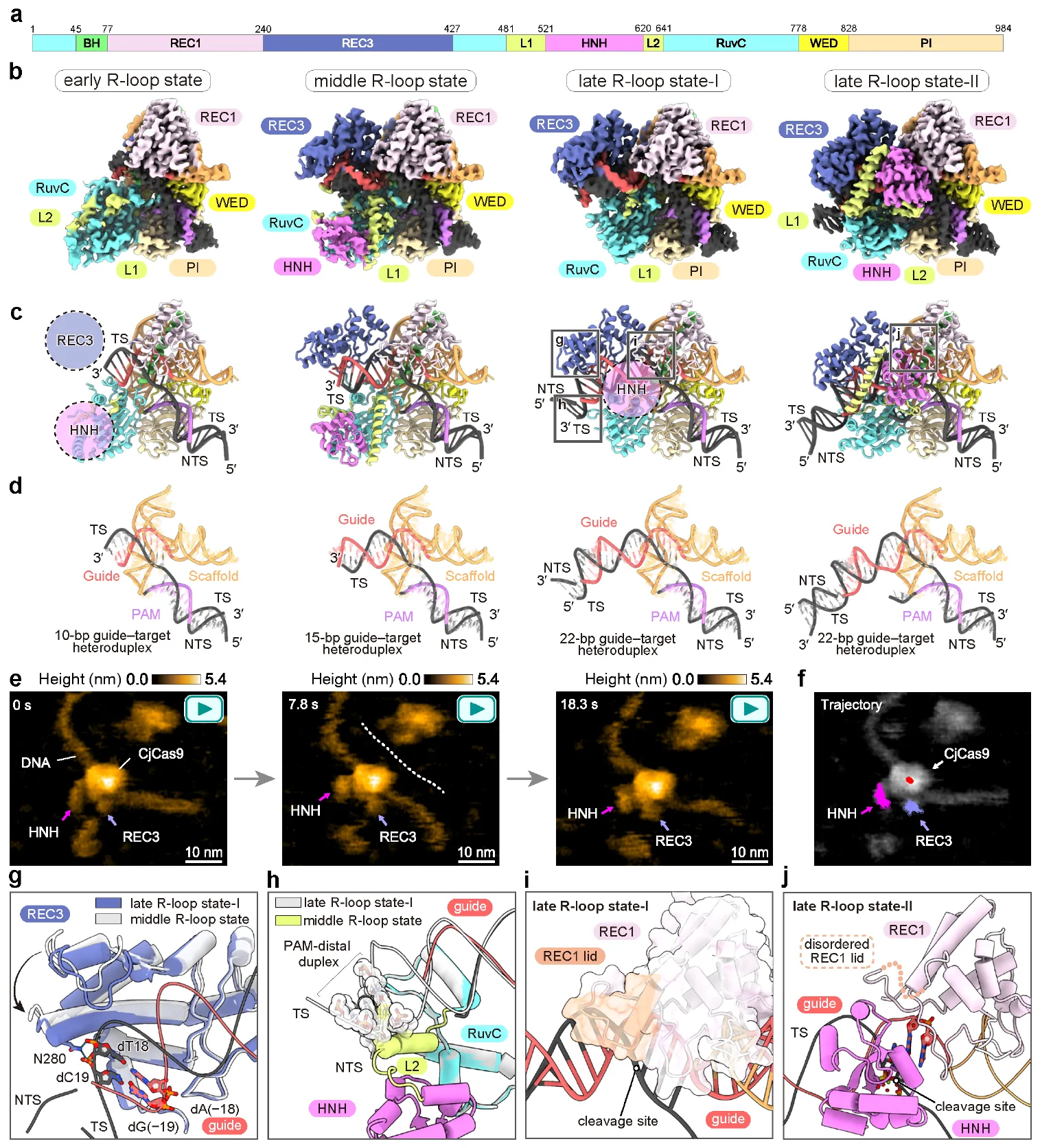
Cryo-EM structures and HS-AFM images of CjCas9 in distinct R-loop states. (**a**) Schematic representations of the domain architectures of CjCas9. (**b and c**) Overview of the early, middle, late-I, and late-II R-loop states of the CjCas9–sgRNA–target DNA. Cryo-EM density maps (**b**), and ribbon models (**c**). (**d**) Structures of the sgRNA, the TS, and the NTS. (**e**) Sequential HS-AFM images of the CjCas9–sgRNA–target DNA complex with the 10-bp guide (See also Supplementary Movie S3). Imaging parameters: scanning/display area = 60 × 48 nm^2^ (240 × 96 pixels^2^); frame rate = 3.3 frames/s (fps). Color bar indicates height (nm). (**f**) Trajectories of the REC3 and HNH domains of CjCas9 tracked over ~30 s. (**g**) Superimposed view of the REC3 domain in the middle R-loop state (colored white) and the late R-loop state-I (colored as in **b**). An inward shift of the REC3 domain enables stable interactions with the REC3 domain and the PAM-distal region of the guide–target heteroduplex. (**h**) Structural comparison of the PAM-distal region between the middle R-loop state (colored as in **b**), and the late-I R-loop state (colored white). The PAM-distal duplex in the late R-loop state-I clashes with the HNH domain in the middle R-loop state. (**i and j**) Close-up views around the TS cleavage site of the late R-loop state-I (i), and the late R-loop state-II (**j**).

**Figure 4:**
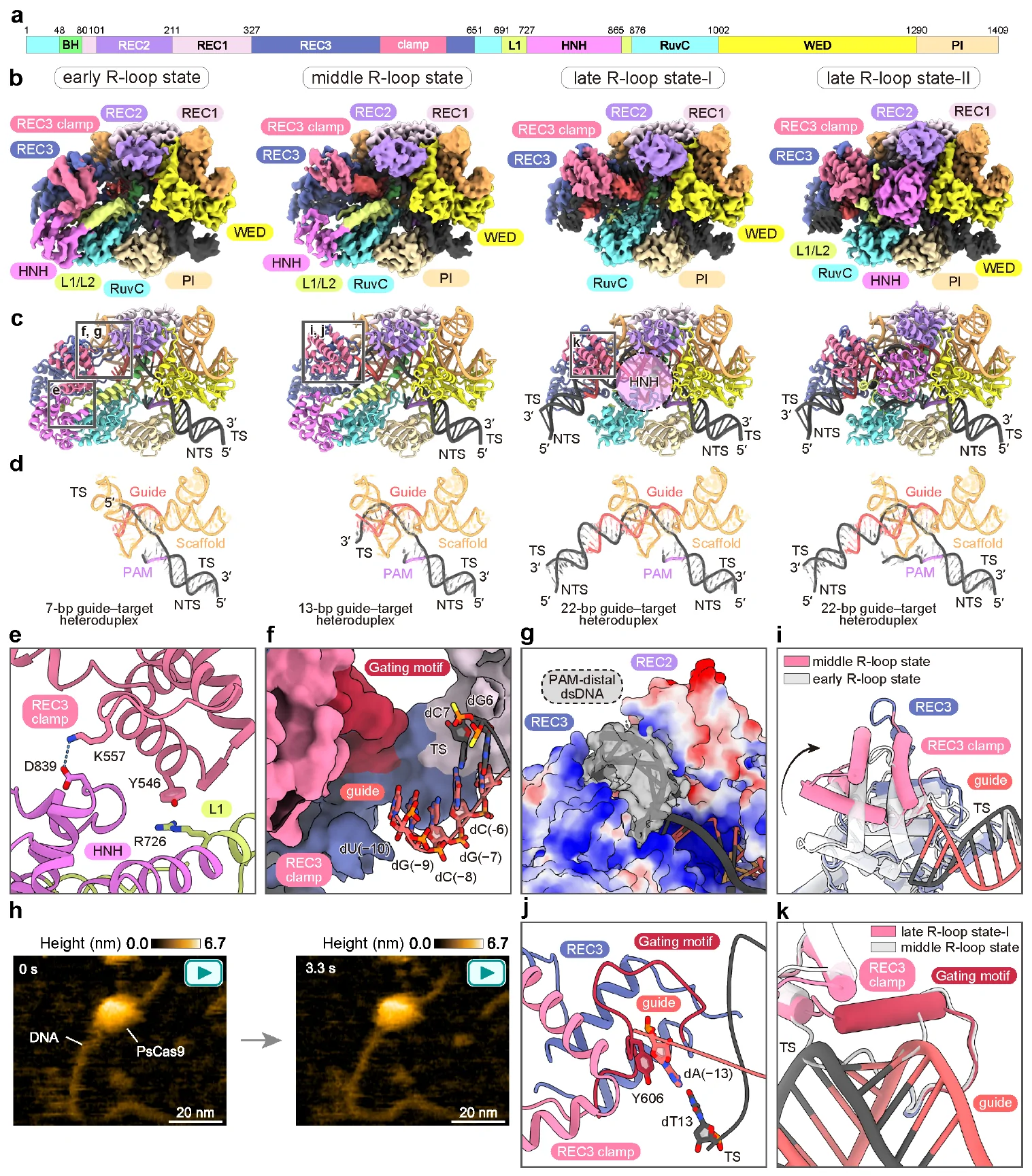
Cryo-EM structures and HS-AFM images of PsCas9 in distinct R-loop states. (**a**) Schematic representations of the domain architectures of PsCas9. (**b and c**) Overview of the early, middle, late-I, and late-II R-loop states of the PsCas9–sgRNA–target DNA. Cryo-EM density maps (**b**), and ribbon models (**c**). (**d**) Structures of the sgRNA, the TS, and the NTS. (**e**) Close-up view of the REC3 clamp interaction in the early R-loop state. The REC3 clamp interacts with the L1 linker and the HNH domain. (**f**) Close-up view of the REC3 gating motif in the early R-loop state. The REC3 gating motif sterically blocks the extension of the guide–target heteroduplex beyond 7 base pairs. (**g**) An ambiguous density corresponding to the PAM-distal dsDNA and surface electrostatic potential map around the REC2 and REC3 domains in the early R-loop state. (**h**) Sequential HS-AFM images of the PsCas9–sgRNA–target DNA complex with the 10-bp guide (See also Supplementary Movie S6). Imaging parameters: scanning/display area = 60 × 48 nm^2^ (240 × 96 pixels^2^); frame rate = 3.3 frames/s (fps). Color bar indicates height (nm). (**i**) Structural comparison of the REC3 domain in the early R-loop state (colored white), and the middle R-loop state (colored as in **b**). An outward displacement of the REC3 clamp allows the extension of the R-loop formation. (**j**) Close-up view of the REC3 gating motif in the middle R-loop state. Tyr606 within the gating motif stacks onto A(−13) in the sgRNA. (**k**) Structural comparison of the REC3 gating motifs between the middle R-loop state (colored white), and the late R-loop state-I (colored as in **b**). The REC3 gating motif shifts outward to create sufficient space required for the completion of the fully matched 22-bp heteroduplex.

**Figure 5:**
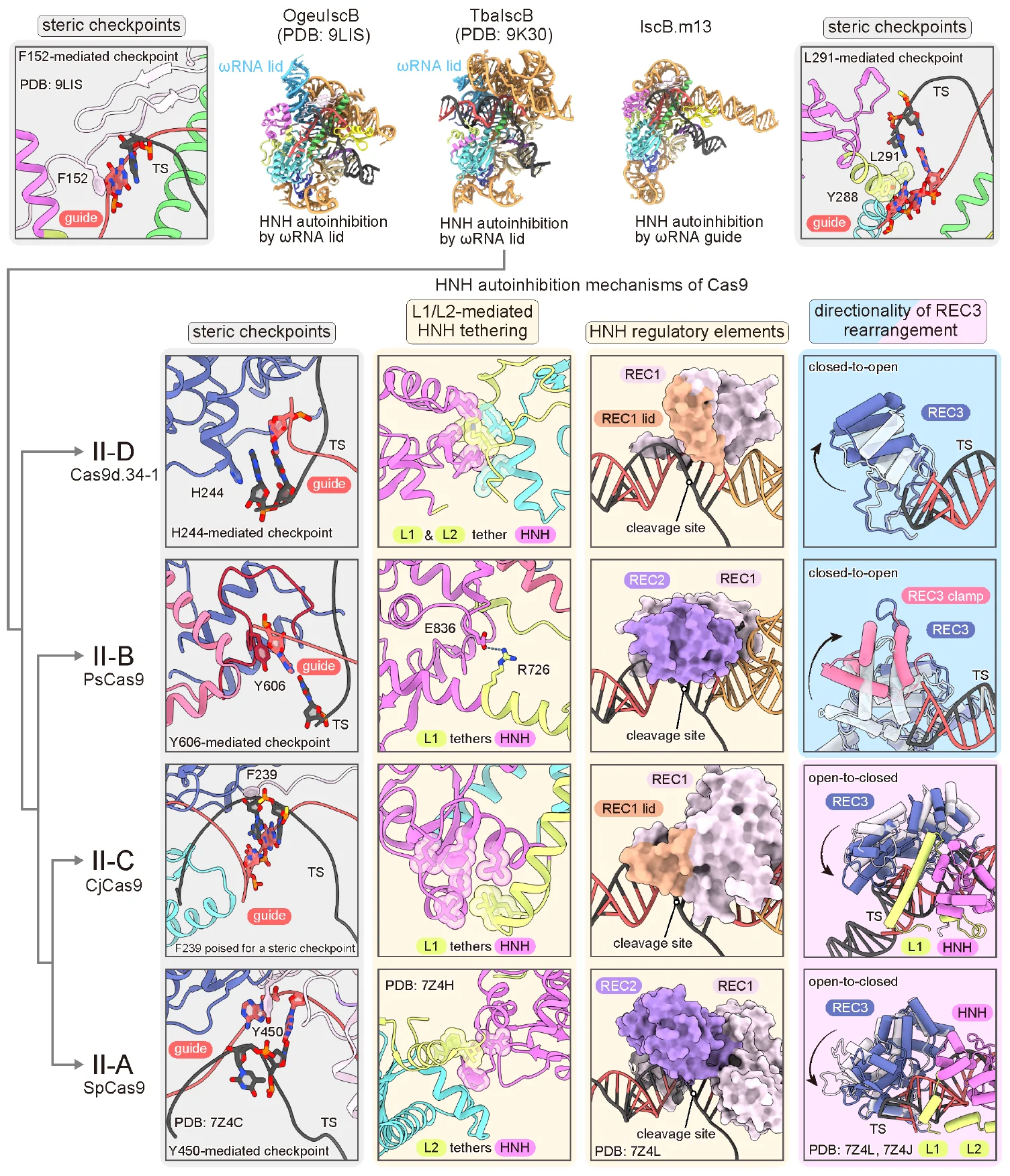
Overview of conserved and diversified nuclease activation mechanisms in the evolution of CRISPR-Cas9. Structural comparison of OgeuIscB (PDB 9LIS), TbaIscB (PDB 9K30), IscB.m13, Cas9d.34-1, PsCas9, CjCas9, and SpCas9 (PDBs 7Z4C, 7Z4H, 7Z4L and 7Z4J). The models at the top represent the two modes of HNH autoinhibition in ancestral IscB-family proteins. OgeuIscB employs the ωRNA lid (colored light blue) to anchor the HNH domain in an inactive position and occlude its active site. TbaIscB, an evolutionary link between IscB and Cas9, also harbors an ωRNA lid analogous to that of OgeuIscB (colored light blue). IscB.m13, which lacks an ωRNA lid, employs the ωRNA guide to maintain the HNH domain in an inactive position. Gray-shaded panels illustrate structurally conserved steric checkpoints across IscB and Cas9 orthologs. A hydrophobic or aromatic residue sterically blocks further extension of the PAM/TAM-proximal guide–target heteroduplex in an early phase of R-loop formation. Yellow-shaded columns depict HNH autoinhibition mechanisms acquired during the evolution of Cas9 from IscB. The left-hand column shows L1/L2-mediated HNH tethering. Cas9 nucleases universally employ the L1 and L2 linkers to tether the HNH domain prior to HNH activation, whereas IscB nucleases employ the ωRNA to anchor the HNH domain. The right-hand column shows the HNH regulatory elements of Cas9 orthologs. Cas9 nucleases have acquired a dedicated protein element that occludes the TS cleavage site to prevent premature HNH docking. In Cas9d.34-1 and CjCas9, the REC1 lid in the REC1 domain occludes the cleavage site until late in R-loop propagation. In PsCas9 and SpCas9, the acquired REC2 domain sterically blocks HNH access to the cleavage site and shifts outward upon HNH activation. Blue-and pink-shaded panels illustrate the two directionalities of REC3 rearrangement during activation. The REC3 domains of Cas9d.34-1 and PsCas9 undergo a closed-to-open transition, thereby opening a path for further guide–target heteroduplex extension (blue). By contrast, the REC3 domains of CjCas9 and SpCas9 undergo an open-to-closed transition, thereby initiating the conformational changes leading to HNH activation (pink).

## Data Availability

The atomic models of the IscB.m13–ωRNA–target DNA complexes have been deposited in the Protein Data Bank under the accession codes 27PP (pre-R-loop state), 27PQ (early R-loop state), and 27PR (late R-loop state). The cryo-EM density maps have been deposited in the Electron Microscopy Data Bank under the accession codes EMD-81326 (pre-R-loop state), EMD-81326 (early R-loop state), and EMD-81326 (late R-loop state). The atomic models of the Cas9d.34-1–sgRNA–target DNA complexes have been deposited in the Protein Data Bank under the accession codes 27OQ (early R-loop state), 27OS (middle R-loop state-I), 27OT (middle R-loop state-II), 27OV (middle R-loop state-III), 27PA (late R-loop state-I), 27PC (late R-loop state-II), and 27PD (late R-loop state-III). The cryo-EM density maps have been deposited in the Electron Microscopy Data Bank under the accession codes EMD-81305 (early R-loop state), EMD-81307 (middle R-loop state-I), EMD-81308 (middle R-loop state-II), EMD-81311 (middle R-loop state-III), EMD-81314 (late R-loop state-I), EMD-81316 (late R-loop state-II), and EMD-81317 (late R-loop state-III). The atomic models of the CjCas9–sgRNA–target DNA complexes have been deposited in the Protein Data Bank under the accession codes 27PJ (early R-loop state), 27PK (middle R-loop state), 27PL (late R-loop state-I), and 27PM (late R-loop state-II). The cryo-EM density maps have been deposited in the Electron Microscopy Data Bank under the accession codes EMD-81321 (early R-loop state), EMD-81322 (middle R-loop state), EMD-81323 (late R-loop state-I), and EMD-81324 (late R-loop state-II). The atomic models of the PsCas9–sgRNA–target DNA complexes have been deposited in the Protein Data Bank under the accession codes 27PU (early R-loop state), 27PV (middle R-loop state), 27PW (late R-loop state-I), and 27PX (late R-loop state-II). The cryo-EM density maps have been deposited in the Electron Microscopy Data Bank under the accession codes EMD-81331 (early R-loop state), EMD-81332 (middle R-loop state), EMD-81333 (late R-loop state-I), and EMD-81334 (late R-loop state-II).

## Code availability

This study did not generate new code.
